# The Impact of SO_2_ Emissions Trading Scheme on Firm’s Environmental Performance: A Channel from Robot Application

**DOI:** 10.3390/ijerph192416471

**Published:** 2022-12-08

**Authors:** Jian Song, Yijing Wang, Jing Wang

**Affiliations:** School of Economics, Nanjing Audit University, Nanjing 211815, China; s_ongking@nau.edu.cn (J.S.); mg2106231@stu.nau.edu.cn (Y.W.)

**Keywords:** emissions trading scheme, environmental performance, robot application, China

## Abstract

Improving the environmental performance of enterprises is the key to achieving the goal of energy conservation, emission reduction and green development. This paper investigates the causal impact on the firm’s environmental performance of China’s SO_2_ emissions trading scheme (SO_2_ ETS), a market-based environmental regulation. Different from the verification mechanism of the Porter hypothesis in the existing literature, we examine the micro mechanism of both emission reduction and efficiency gains of enterprises from the perspective of robot application based on Chinese firm-level data from 2000 to 2013. The paper found that SO_2_ ETS significantly reduces the emission intensity of Chinese enterprises, and the results are still significant after a series of robustness tests and using instrumental variables to overcome the endogeneity problem. Mechanism analysis shows that the reduction of pollutant emissions and the productivity effect of robot application are two significant channels for SO_2_ ETS to improve the firm’s environmental performance. In addition, in resource-based and recession-oriented cities, the SO_2_ ETS has a more significant effect on enterprise emission reduction. These findings provide empirical evidence and policy enlightenment for enterprises to promote market-oriented environmental regulation and release institutional dividends in the process of industrial automation transformation, green and sustainable development.

## 1. Introduction

Controlling emissions has become a common concern in many countries. Since the reform and opening-up, China’s economy has been growing at a high speed, but environmental deterioration and ecological pollution are becoming increasingly serious. As the largest developing country and the largest emitter of sulfur dioxide (He, 2010 [1]), China attaches great importance to the emissions of pollutants, constantly learns from the useful practices of other countries, and takes measures to reduce sulfur dioxide emissions. Historically, the American economist Dales proposed in 1968 that emission trading was an important market-based environmental regulation. The earliest practice was the sulfur dioxide trading system established by the Clean Air Act Amendment in 1990. Subsequently, in 2005, the 27 members of the European Union established the world’s first multinational EU-Emissions Trading System (EU-EST), which is also the world’s largest cap-and-trade system for carbon emissions. The establishment of EST is of great significance for reducing pollutant emission and protecting the environment, and its beneficial experience is worthy of China’s attention and reference.

For China, in the early stage, the government primarily implemented command-based environmental laws and regulations, and began exploring SO_2_ ETS in the 20th century. In the early 21st century, market-based environmental regulations were gradually implemented, and in 2007, the Chinese government officially launched a pilot SO_2_ ETS in 11 provinces. The government has since favored trading emissions permits as a commodity to reduce emissions. At present, the SO_2_ ETS has become an important means to control pollution and protect the environment in China.

Existing environmental regulations can be divided into two categories: command-based environmental regulations (Lanoie et al., 2008 [2]; Rubashkina et al., 2015 [3]; Chen et al., 2021 [4]) and market-motivated environmental regulations represented by the emissions trading mechanism and carbon tax (Carlson et al., 2000 [5]; Franco and Marin, 2017 [6]). Compared to the command-based environmental regulation that forces enterprises to adopt unified pollution reduction measures, market-motivated environmental regulation allows enterprises to buy or sell emission rights on demand. Therefore, enterprises choose their own emissions, according to the marginal cost of pollution reduction reduce, which can achieve less impact on the business environment while reducing the emission of pollutants (Hu et al., 2020 [7]). Under the strict supervision and management of the government, the stipulated emissions ceiling can effectively reduce emissions in the pilot provinces (Li and Shen, 2008 [8]). As the micro subjects of economic activities, enterprises are the main constraint object of SO_2_ ETS. The performance of enterprises in SO_2_ ETS can better measure whether this environmental regulation is effective. Therefore, after clarifying the effectiveness of emissions trading regulations on pollution reduction, we want to further explore how SO_2_ ETS affect emissions behavior of enterprises. How will enterprises react to the policy shock? Do SO_2_ ETS reduce enterprises’ emissions or the intensity of their emissions? Will companies reduce their emissions at the expense of their own interests, such as reducing production? To answer the above questions, this paper explores how the SO_2_ ETS affects the environmental performance of enterprises, which is reflected by the emissions intensity of pollutants (Liu and Chen, 2020 [9]; Su and Sheng, 2021 [10]).

Additionally, the SO_2_ ETS mechanism of corporate environmental performance is often the focus of attention. SO_2_ ETS increases the environmental cost of enterprises (Sijm et al., 2006 [11]; Fabra and Reguant, 2014 [12]). On the one hand, it forces enterprises to reduce emissions; on the other hand, it may cause enterprises to adjust production (Albrizio et al., 2017 [13]). According to existing studies, scholars primarily focus on the “Porter hypothesis” (Porter, 1995 [14]) to investigate whether environmental regulations can achieve environmental protection and economic development through technological innovation (Dechezlepretre et al., 2019 [15]). However, in addition to technological innovation, the absorption of advanced production technologies through the adoption of advanced intelligent production equipment has forged an important path for China’s technological progress and productivity improvement (Shi and Li, 2019 [16]; Wan et al., 2021 [17]), particularly for enterprises with insufficient R&D capabilities (Shao et al., 2020 [18]). The adoption of intelligent production characterized by robots has led to a new round of production changes in many countries. The widespread use of robots is particularly evident in China, where the stock of robots increased from 930 to 7.83 million between 2000 and 2019, according to the International Federation of Robotics. The widespread application of robots in China has had a great impact on the production activities of Chinese enterprises. The application of robots in production can reduce labor costs and improve production technology and productivity (Graetz and Michaels, 2018 [19]). In this context, it is worth discussing the practical issue of whether SO_2_ ETS can affect the emissions and productivity of enterprises through the application of robot production so as to improve the environmental performance of enterprises.

Therefore, based on the perspective of robot application and combined with microlevel enterprise emissions data, this paper tests the policy effect and mechanism of SO_2_ ETS on enterprises’ environmental performance on theoretical and empirical levels. Specifically, we first introduced robot production factors and the emissions trading market into the heterogeneous enterprise model of Melitz (2003) [20] to investigate the internal mechanism of SO_2_ ETS affecting enterprise emissions intensity. Combined with the matching data of the China Industrial Enterprise Database, the China Industrial Pollution Source Key Investigation Enterprise Database, and the Customs Database from 2000 to 2013, the pilot policy of sulfur dioxide emissions trading in 2007 was used as a quasi-natural experiment, and the difference-in-differences method was used to identify the impact of SO_2_ ETS on enterprise environmental performance. This study finds that under the rigid pressure of SO_2_ ETS, enterprises reduce the emissions intensity of pollutants (thereby improving their environmental performance) by reducing emissions and improving enterprise productivity through the application of robot production.

Compared with the existing literature, this paper makes the following three contributions. First, unlike most literature that examines the effectiveness of SO_2_ ETS from the macro perspective, this paper discusses the impact of the system from the microenterprise level and uses emissions intensity to represent the level of enterprise environmental performance to explore the impact of emissions trading policy on enterprise environmental performance. This paper complements research on environmental rights trading programs. Second, unlike the technological innovation mechanism emphasized by the “Porter hypothesis,” we explore the action mechanism of SO_2_ ETS on the enterprise environmental performance from the perspective of the productivity effect of robots based on the characteristics of a large number of robots used in China, which adds a new path to few studies. Third, the current research on robots largely comes from the evidence of developed countries, while the research on developing countries primarily discusses the economic effects of robot application, and few studies explore the internal reasons for the increase in robots. This paper believes that environmental regulations can increase investment in robots and that robots are effective in reducing emissions and improving production efficiency. This conclusion enriches the related research on robots.

## 2. Background Literature

### 2.1. Related Research on the Emissions Trading Mechanism

The economic effect brought by environmental regulation has long been a hot topic in the field of environmental economics. The pilot policy of emissions trading is a type of environmental regulation that attracts much attention. Emission trading originated in the United States. In 2005, the European Union established the EU-ETS, which has become the largest carbon emission trading market in the world and plays an exemplary role in the world. Early studies on the emission trading mechanism mainly focused on the United States and the European Union. For example, Cason and Plott (1996) [21] confirmed the effectiveness of the SO_2_ ETS in the United States based on laboratory data. Hoffmann (2007) [22] made an empirical analysis of the EU-ETS through the dynamic panel model and believed that there were two reasons for the reduction of pollutant emissions: the emission reduction achieved by emission rights and the emission reduction achieved by the 2008 economic crisis. Schleich et al. (2009) [23] found in the study of the European Union that although the effect of EU-ETS on the improvement of energy efficiency is limited in the current period, there is still a huge space in the future. Anderson et al. (2010) [24] studied the impact of EU-ETS on CO_2_ emission reduction in manufacturing and found that EU-ETS can also encourage enterprises to carry out environmental innovation.

With the deepening of scholars’ research, the research on the pilot policy of emission trading is gradually conducted on aspects such as policy effects, economic performance (Rubashkina et al., 2015 [3]; Albrizio et al., 2017 [13]) and technological innovation (Calel and Dechezlepretre, 2016 [25]; Dechezlepretre et al., 2019 [15]; Wang et al., 2021 [26]). On the one hand, the research conclusions of some scholars support the emissions reduction effect of the emissions trading mechanism. For example, Chan et al. (2013) [27] analyzed that EU-EST can effectively reduce pollutant emissions. Ren et al. (2019) [28] evaluated the policy effects of China’s carbon emissions trading mechanism and found empirical evidence from both provinces and prefectural-level cities that showed the emissions trading pilot system could effectively reduce emissions. Alternatively, some scholars believe that the effects of emission rights policies are limited. For example, Borghesi et al. (2015) [29] used panel data on Italian manufacturing industries and found that the policy effect of the European emissions trading scheme was limited owing to loose quota issuance. Bel and Joseph (2015) [30] used dynamic panel data to evaluate the EU ETS and believed that the emissions reduction effect of ETS was very limited.

In recent years, researchers have gradually realized the lack of research on enterprise-level issues from environmental regulation policies and have begun to analyze micro subjects based on data from developed countries. Research on the impact of environmental regulation on a microlevel involves innovation input (Kneller and Manderson, 2012 [31]; Huang et al., 2021 [32]), total factor productivity (Ryan, 2012 [33]; Chen et al., 2021 [4]), employment (Walker, 2011 [34]; Curti, 2018 [35]), and enterprise competitiveness (Hering and Poncet, 2014 [36]; Liu et al., 2021 [37]). While only a small amount of literature on the environmental regulation intensity level of enterprise emissions and the role of channels, those such as Zhang et al. (2020) [38] argue that permits to increase the cost of emissions lower coal consumption or stimulate enterprises to innovate to improve coal utilization efficiency and reduce emissions; Fan et al. (2019) [39] empirically verified that polluting enterprises primarily increase investment in recycling and emissions reduction equipment to meet stricter environmental regulations.

### 2.2. Related Research on Robot Application

Although the use of robots is increasingly widespread worldwide, there is little international literature that directly studies the impact of robots on enterprise productivity. Existing literature has shown through empirical analysis at the national level that technological progress can improve production efficiency and drive overall economic development. For example, Aly (2020) [40] verified that intelligent development can promote economic development and labor productivity by using cross-sectional data of 25 developing countries in 2017. Graetz and Michaels (2018) [19] analyzed the data of 17 developed countries and found that robot input increased the total factor productivity of manufacturing enterprises by 0.36%. In industrial research, some scholars have confirmed that the use of robots can improve productivity (Acemoglu and Restrepo, 2020 [41]). For example, Li et al. (2020) [42] pointed out that from 2003 to 2015, China’s manufacturing total factor productivity grew at an average compound annual rate of 8.2%, and much of this was driven by technological advancements, exemplified by the increased use of robots. Further, some scholars analyze enterprise-level data and point to robot applications can promote productivity. For example, Graetz and Guy (2018) [19] believe that robots are beneficial to alleviate the problem of unreasonable task allocation in R&D departments of enterprises, and can monitor the progress of R&D tasks in real time to avoid inefficiency caused by disorder. Li and Xu (2020) [43] analyzed the data of Chinese industrial enterprises and concluded that the productivity of manufacturing enterprises using robots would be 7.45% higher than that of enterprises without robots. Fu and Lv (2022) [44] used the national survey data of the Chinese Academy of Social Sciences to find that industrial robots have a positive effect on improving the current and expected production performance of enterprises.

Additionally, the relationship between industrial robots and green production behavior of enterprises is a new topic that needs to be studied urgently in the field of environmental economics. Although not much literature directly explores the relationship between the two, existing literature has confirmed that technological upgrading methods such as technological progress and technological innovation can improve the green production level of enterprises (Xu et al., 2020 [45]; Singhania and Saini, 2021 [46]). In the early stage, Grossman and Krueger (1995) [47] argued in their pioneering theoretical framework that technological progress and the use of clean technologies would reduce emissions of enterprises. Singhania and Saini (2021) [46] using emissions data from 21 countries from 1990 to 2016, found that technological innovation effects in developing countries can presently inhibit environmental degradation. Fang (2021) [48] found in the environmental effect assessment of China’s high-speed rail operation that the technological progress brought by high-speed rail is conducive to reducing soot pollution in cities along the route.

In summary, there are the following deficiencies existing in the literature: (1) Market-oriented environmental regulation of most studies focuses on developed economies. Few empirical studies come from developing countries. The study of China’s emissions trading scheme is very limited. In addition, there is a lack of research on the impact of market-based environmental regulations on pollutant emission intensity at the micro enterprise level. (2) Existing studies primarily focus on how the rise of robots affects the labor market in developed economies such as Europe and the United States, while evidence from developing countries is still relatively lacking. Moreover, previous literature focused on the impact of robot application on the labor market, and not much literature investigated what factors led to the application of industrial robots. (3) The existing literature has confirmed that technological progress can promote the green production of enterprises. In the context of the large number of robots introduced into production, not much literature explores the green production behavior of enterprises from the perspective of robot application. This paper reveals that the application of robots is a significant way for the ETS to improve the environmental performance of enterprises, which is a supplement to the existing research on the ETS. Based on this, this paper takes the 2007 pilot policies as the research object and examines the impact of SO_2_ ETS on the environmental performance of Chinese enterprises from the perspective of robot application.

## 3. Theoretical Framework and Hypotheses

To construct the micro mechanism of the SO_2_ ETS on enterprise environmental performance, we try to make the following expansion based on the heterogeneous enterprise model constructed by Melitz (2003) [20]: (1) Introduce the enterprise application of robot production behavior and robot application can improve the level of enterprise productivity (Acemoglu and Restrepo, 2019 [49]); (2) Construct the emissions trading market, and consider the efficiency of the emissions trading market and the environmental regulation pressure imposed by the government.

### 3.1. Assumptions of the Model

Based on the utility function of constant elasticity of substitution (CES), it is assumed that the representative consumer’s preference for differentiated products is in the form of CES utility function as the following:

(1) $$U={\left[\int_{i\in I}q{\left(i\right)}^{\frac{\sigma -1}{\sigma }}di\right]}^{\frac{\sigma }{\sigma -1}}$$

where q(i) represents consumers’ demand for differentiated products, and σ(σ>1) represents the elasticity of substitution between differentiated products. According to the principle of utility maximization, the demand function of consumers for differentiated products can be deduced as follows:

(2) $$q(i)=\frac{p{\left(i\right)}^{-\sigma }}{P^{1-\sigma }}R$$

where p(i) represents the market price of differentiated products; $P={\left(\int_{i\in I}p{\left(i\right)}^{1-\sigma }di\right)}^{\frac{1}{1-\sigma }}$ represents the total price index of differentiated products; and $R=\int_{i\in I}p(i)q(i)di$ denotes the total consumer payment for all goods.

The assumptions of the heterogeneous firm model are as follows: (1) Firm heterogeneity is determined by initial productivity $\varphi_{i0}$ ( $\varphi_{i0}>0$ ). (2) Enterprises can choose whether to invest in the production of robots. The input of robots is γ(i) ( γ(i)≥0 ), and the application of robots can improve the productivity of enterprises to $\varphi (i)=\varphi_{i0}e^{\gamma (i)}$ . The price of a single robot is fixed as c so the cost for enterprises to purchase robots is cγ(i) . (3) The amount of energy input is s(i) , and the output q(i) is a linear function of energy input and productivity, namely: q(i)=s(i)φ(i) . (4) The energy price is $p_{s}$ , so the cost for the enterprise to purchase energy is $s(i)p_{s}=p_{s}q(i)/\varphi (i)$ . (5) The government gives each enterprise a quota of emission rights T(i) , and the balance of aggregate supply and aggregate demand determine the trading price of emission rights $p_{t}$ .

The assumptions of the ETS on the constraints of enterprise behavior are as follows: (1) Enterprises must abide by the ETS regulations and must not discharge pollutants beyond the scope of emission rights they have; (2) The EST enables emission rights to be purchased and sold. The parameter μ(0≤μ≤1) is introduced to represent the operation efficiency of the emission rights market, indicating that only μ proportion of pollutants can be discharged through the purchase of emission rights. The cost for the enterprise to purchase the emissions right is $p_{t}[\frac{\mu q(i)}{\varphi (i)}-T(i)]$ .

Based on the above assumptions, the profit function of an enterprise aiming for profit maximization is as follows:

(3) $$Max\;\pi (i)=p(i)q(i)-\left[f_{e}+c\gamma (i)+s(i)p_{s}\right]-p_{t}\left[\frac{\mu q(i)}{\varphi (i)}-T(i)\right]\;s.t.\;\frac{\mu q(i)}{\varphi (i)}-T(i)\leq 0$$

The constraint conditions represent the government’s control behavior—that is, except for the pollutants in proportion μ that can be traded, the emissions of the remaining pollutants shall not exceed the emissions quota of the enterprise.

When constraints are tight and Lagrange multipliers are introduced, Equation (3) can be written as follows:

(4) $$Max\;L\left(i\right)=\left[p(i)-\frac{p_{s}+\mu p_{t}+(1-\mu )\lambda (i)}{\varphi_{i0}e^{\gamma (i)}}\right]q(i)-f_{e}-c\gamma (i)+\left[p_{t}+\lambda (i)\right]T(i)$$

### 3.2. Spatial Equilibrium/Equilibrium in a Closed Economy

After substituting Equation (2) into Equation (3), the derivative of p(i) and γ(i) is obtained as the following:

(5) $$\frac{\partial \pi (i)}{\partial p(i)}=\frac{p{\left(i\right)}^{-\sigma }}{P^{1-\sigma }}R\left\{1-\sigma \left[p(i)-\frac{p_{s}+\mu p_{t}+(1-\mu )\lambda (i)}{\varphi_{i0}e^{\gamma }}\right]p{\left(i\right)}^{-1}\right\}=0$$

(6) $$\frac{\partial \pi (i)}{\partial \gamma (i)}=\frac{p_{s}+\mu p_{t}+(1-\mu )\lambda (i)}{\varphi_{i0}e^{\gamma (i)}}\times \frac{p{\left(i\right)}^{-\sigma }}{P^{1-\sigma }}R-c=0$$

After combining Equations (5) and (6), the commodity price, output, and robot input when enterprise profit maximization is achieved as the following:

(7) $$p^{*}(i)=P{\left(\frac{\sigma -1}{\sigma }\times \frac{R}{c}\right)}^{\frac{1}{\sigma -1}}$$

(8) $$q^{*}(i)=\frac{1}{P}{\left[\frac{c\sigma }{\sigma -1}\times {\left(\frac{1}{R}\right)}^{\sigma }\right]}^{\frac{1}{\sigma -1}}$$

(9) $$\gamma^{*}(i)=\ln\left\{\frac{1-\mu }{T(i)}\times \frac{1}{P\varphi_{i0}}{\left[\frac{c\sigma }{\sigma -1}\times {\left(\frac{1}{R}\right)}^{\sigma }\right]}^{\frac{1}{\sigma -1}}\right\}$$

It is assumed that emissions g(i) is proportional to the amount of energy input—that is, g(i)=ks(i)=kq(i)/φ(i) . Therefore, the expression of emissions g(i) and emissions intensity h(i) can be calculated as the following:

(10) $$g(i)=\frac{kq(i)}{\varphi (i)}=\frac{kT(i)}{1-\mu }$$

(11) $$h(i)=\frac{w(i)}{q^{*}(i)}=\frac{k}{\varphi (i)}=\frac{kPT(i)}{1-\mu }{\left[\frac{\sigma -1}{c\sigma }\times R^{\sigma }\right]}^{\frac{1}{\sigma -1}}$$

### 3.3. Research Hypotheses

Before the implementation of the emissions trading policy, enterprises were not allowed to trade emission rights, in which case μ=0 ; however, when the policy is implemented, μ∈(0,1] . The government’s control of emissions is reflected in the emission rights quota T(i) ; the greater the government’s pollution reduction, the smaller emission rights quotas T(i) enterprises have, and the emission rights that can be traded decrease accordingly. To explore the impact of emissions trading pilot policies on enterprise environmental performance, the derivative of emissions intensity in Equation (11) with respect to T(i) is obtained as the following:

(12) $$\frac{dh(i)}{dT(i)}=\frac{kP}{1-\mu }{\left[\frac{\sigma -1}{c\sigma }\times R^{\sigma }\right]}^{\frac{1}{\sigma -1}}$$

where 1−μ>0 and σ−1>0 , so dh(i)/dT(i)>0 . The above equation shows that the stricter the emissions trading pilot policy, the smaller the emission rights quota T(i) owned by the enterprise, which will reduce its emissions intensity and thus improve the environmental performance of the enterprise. The following is the conclusion:

**Hypothesis** **1.** *SO_2_ ETS can improve the environmental performance of enterprises.*

Additionally, we not only want to know the direction of action of the emissions trading mechanism on corporate environmental performance, we also want to explore the mechanism of action. According to the expression h(i)=w(i)/q(i) of emissions intensity, it can be seen that pollutant emissions quantity determines pollutant emissions intensity. Therefore, by taking the derivative of emissions in Equation (10) with respect to T(i) , we can obtain the following:

(13) $$\frac{dw(i)}{dT(i)}=\frac{k}{1-\mu }>0$$

The above equation shows that the smaller the emission rights quota of an enterprise, the fewer total pollutants it emits. Therefore, the emission rights trading pilot policy can reduce the emissions and then reduce the emissions intensity. The following is the conclusion:

**Hypothesis** **2.** *SO_2_ ETS promote the environmental performance of enterprises by reducing the emissions of pollutants, thus reducing the emissions intensity of pollutants.*

Furthermore, the expression of emissions intensity in this paper can also be written as h(i)=k/φ(i) , indicating that there is a negative correlation between productivity and emissions intensity. Barrows and Ollivier (2018) [50] confirmed the negative correlation between enterprise productivity and emissions intensity using theoretical models. Shapiro and Walker (2018) [51] also verified the negative correlation between enterprise productivity and pollution emissions per unit of labor force. Based on this, we believe that the improvement of productivity can reduce the emissions intensity of enterprises. Because robot applications can increase enterprise productivity to $\varphi (i)=\varphi_{i0}e^{\gamma (i)}$ , robot applications can improve environmental performance by increasing enterprise productivity. If the pilot emissions trading policy can affect the use of robots, the policy can affect the environmental performance of enterprises through the aforementioned channels. To explore the influence of the emissions trading pilot policy on robot application, the robot expression in Equation (9) is differentiated with respect to T(i) , and the following results are obtained as the following:

(14) $$\frac{d\gamma (i)}{dT(i)}=-\frac{1}{T(i)}<0$$

The above equation shows that the reduction of emission rights quotas will promote enterprises to invest in robot production—that is, the emissions right trading mechanism can promote enterprises to utilize robots. Therefore,

**Hypothesis** **3.** *SO_2_ ETS can improve enterprise productivity and environmental performance by increasing enterprise investment in robots.*

**Figure 1:**
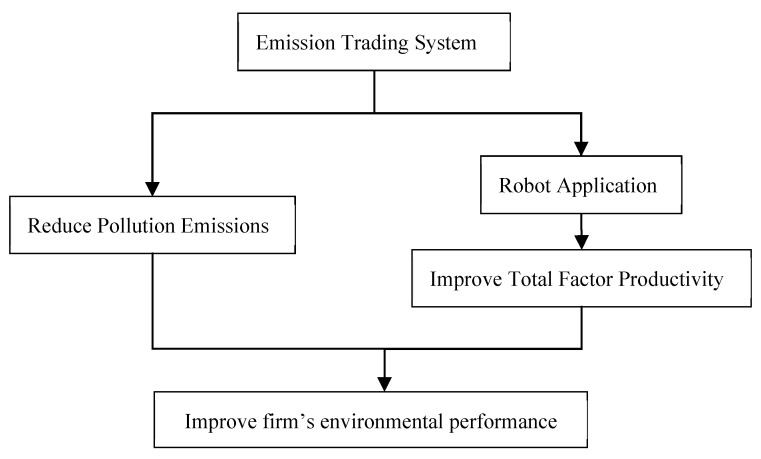
Mechanism diagram of the impact of the emissions trading mechanism on corporate environmental performance.

According to the above analysis, the micro mechanism diagram of the impact of the emissions trading mechanism on enterprise environmental performance can be obtained as shown in Figure 1.

## 4. Empirical Strategy and Data

### 4.1. Econometric Model

In the empirical analysis section, this paper takes the 2007 pilot policy of s SO_2_ ETS as a policy shock and tests the above theoretical analysis results by evaluating its policy effects. The Difference-in-Differences (DID) method can effectively separate the “policy disposition effect” and estimate the net impact of a policy on participants, so it is widely used for the evaluation of policy effects. In view of this, the benchmark regression equation is set as follows:

(15) $$\ln Sd_{isct}=\beta_{0}+\beta_{1}time\times treat+\beta_{3}X+\mu_{s}+\eta_{c}+\gamma_{t}+\varepsilon_{isjt}$$

where the subscripts *i*, *s*, *c*, and *t* indicate the enterprise, industry, province, and year, respectively. The explained variable *Sd_isct_* is the SO_2_ emissions intensity of enterprise, which is used to measure enterprise environmental performance. *time* and *treat* are dummy variables with values of 0 and 1. *time* is set to 1 after the emissions trading pilot in 2008; otherwise it is set to 0; *treat* is set to 1 if the enterprise is located in the pilot area; otherwise it is set to 0. *X* is a set of control variables, which primarily control the two levels of enterprise and province, including enterprise age (*Age*), enterprise size (*Scale*), enterprise ownership (*Owner*), asset-liability ratio (*Alr*), capital intensity (*Kl*), regional economic structure (*Se_gdp*), and environmental regulation intensity (*Regulation*). $\delta_{i}$ , $\mu_{s}$ , $\eta_{c}$ , and $\gamma_{t}$ represent firm fixed effects, industry fixed effects, province fixed effects, and time fixed effects.

The DID model is selected for an empirical test, and its basic idea is to regard the implementation of new policies as a “natural experiment” or “quasi experiment” of external economic system. Regarding the implementation of SO_2_ ETS in China; on the one hand, the environmental performance of the same pilot enterprise is different in the implementation of the policy. On the other hand, an index at the same time point is different between the pilot enterprise and the non-pilot enterprise. The model regression estimation based on the above double differences can effectively control the influence of other policies at the same period, as well as the differences between the experimental group and the control group, so as to identify the net impact of policy shocks on corporate environmental performance. In the regression part, we adopt two regression strategies. One is to control the fixed effect of time, industry and province, which can control the characteristics that do not change with time. The second is to further control the individual fixed effect, that is, to use the two-way fixed effects (TWFE DID) model, so that the individual characteristics that do not change with time can be considered more carefully. For the robustness of the empirical results in this paper, the regression results of these two strategies will be listed simultaneously.

### 4.2. Aariables Declaration

The core explained variable is enterprise environmental performance (*Sd*), whose concept can be defined from two aspects. In a broad sense, it refers to the continuous improvement of the comprehensive effect of the enterprise’s pollution control and resource utilization; in a narrow sense, it refers to an indicator that can be directly detected. In this paper, the emission intensity of enterprises is used to reflect the environmental performance of enterprises, which not only corresponds to the theoretical model and research hypothesis, but also can eliminate the influence of scale factors. In particular, the emission intensity of pollutants is more consistent with the national conditions of developing countries than the total emission. For a developing country such as China, it is a more feasible policy goal to seek a green improvement of production while maintaining the economic growth rate. Considering that SO_2_ ETS mainly controls SO_2_ emission, SO_2_ emission intensity is selected in this paper to reflect the environmental performance level of enterprises.

The measurement of other variables is shown in Table 1.

### 4.3. Data

**Figure 2:**
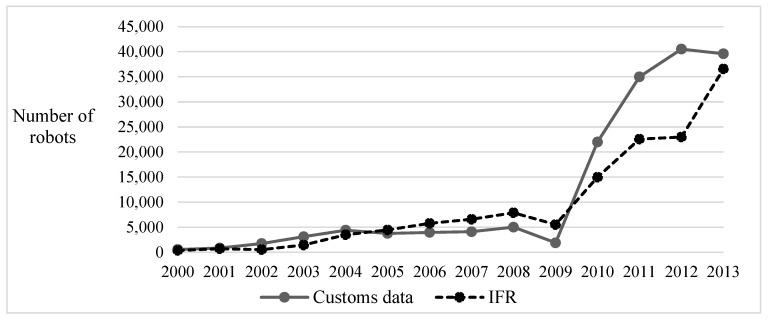
Comparison of the number of industrial robots used and imported in China.

This paper uses the matching data of the China Industrial Enterprise Database, the China Industrial Pollution Source Key Investigation Enterprise Database, and the Customs Database from 2000 to 2013. Among them, the China Industrial Enterprise Database covers the industrial enterprises whose main business incomes reach 5 million yuan or more and develops detailed statistics on basic information such as establishment date and location of the enterprise, as well as financial information such as assets and income. As the most comprehensive and reliable microenvironmental database in China at present (Wang, 2020 [54]), the Database of China’s Key Survey Enterprises on Industrial Pollution Sources records, in detail, the emissions of industrial waste gases and wastewater, such as sulfur dioxide and chemical oxygen demand by polluting enterprises. The reasons for choosing the data of enterprise imported robots in China Customs Data as a proxy variable of robot application are as follows: (1) At present, the authoritative use data of robots come from IFR. Figure 2 shows that the number of customs-imported robots is generally consistent with the number of IFR robot applications, indicating that the import volume of robots can reflect the status of robot applications to a certain extent. (2) IFR data are at the industry level, so the robot application data at the enterprise level are not available. However, China Customs Data provide the HS eight-digit coding information of the product, which can identify the imported number of enterprise robots and can be used as a proxy variable for robot application (Li et al., 2021 [55]). There is much literature that uses robots imported by enterprises as proxy variables for robot applications (Acemoglu and Restrepo, 2018 [56]).

The cleaning and matching process of the data in this paper is as follows. First, we cleaned up the database of Chinese industrial enterprises by deleting the following types of enterprises: those with fewer than 8 employees, those missing major financial information (such as gross industrial output value and total assets information), and those who did not conform to international general accounting standards (such as having fixed assets larger than total assets and or total liabilities fewer than 0). Additionally, because the national industry classification standards were changed in 2002 and 2011 (during the sample period), we uniformly adjusted the industry classification codes for all years to the 2002 National industry classification standards. Second, we matched the database of China industrial enterprises with the database of China Key Investigation enterprises who were sources of industrial pollution according to the legal code. We then matched the unmatched samples based on the method of “enterprise name + administrative code” (Wan et al., 2021 [17]). Finally, we screened the data of imported robots according to the HS 6-digit code and matched the database of Chinese industrial enterprises with the customs database to obtain the database for regression analysis in this paper.

### 4.4. Characteristic Facts

**Figure 3:**
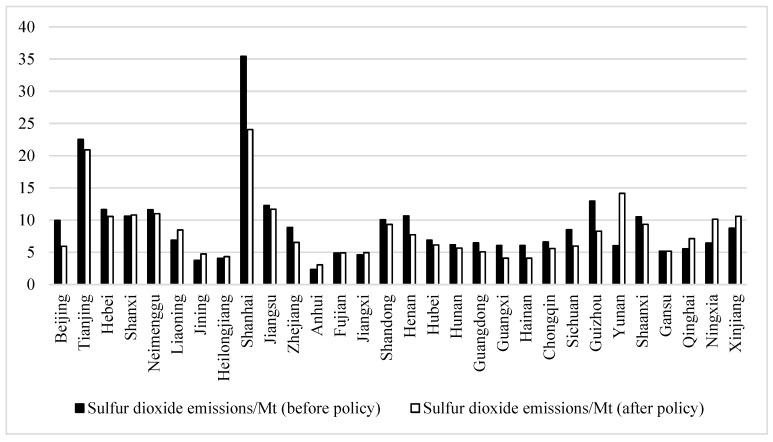
Comparison of SO_2_ emissions before and after the implementation of the policy.

The effective implementation of the SO_2_ ETS is a prerequisite for studying the relationship between the SO_2_ ETS and corporate environmental performance. Therefore, this paper first tests whether the SO_2_ ETS is effective in reducing SO_2_ emissions. Figure 3 shows the mean change of SO_2_ emissions in each region before and after the implementation of the policy. As seen in the Figure 3, emissions decreased in all pilot areas (except Shanxi Province, where sulfur dioxide emissions increased), with the most obvious performance being Shanghai, which indicates that the SO_2_ ETS has an effect on emissions reduction. Additionally, it can be seen that the pilot areas are generally high-pollution areas, indicating that the implementation of the emissions trading mechanism has regional pertinence.

**Figure 4:**
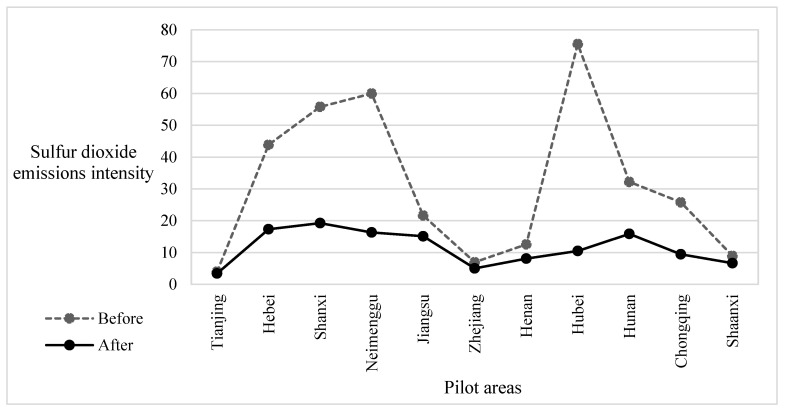
Comparison of emission intensity before and after the pilot policies.

Second, to observe the relationship between the SO_2_ ETS and the environmental performance of enterprises, we draw a comparison chart of the average environmental performance level of enterprises in the pilot areas before and after the implementation of the policy. The mean value of the SO_2_ emissions intensity of pilot areas in the 5 years before (or after) the implementation of the policy is selected to represent the average performance level of enterprises before (or after) the implementation of the policy, which is represented by the dotted line (or the solid lines) in the Figure 4. As shown in Figure 4, the dotted line is always above the solid line, indicating that after the implementation of the policy, the average SO_2_ emissions intensity of enterprises in the pilot area has been reduced, and the environmental performance of enterprises has improved. Therefore, we preliminarily determine that the SO_2_ ETS can improve the environmental performance of pilot areas.

**Figure 5:**
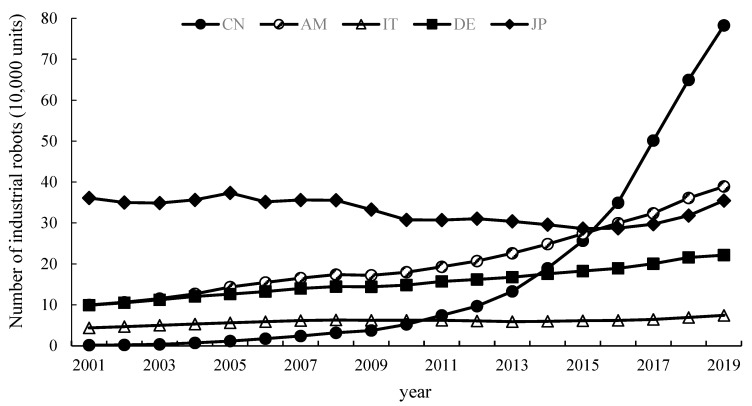
Industrial robot inventory in major countries from 2001 to 2019.

In addition, this paper considers that the application of robots is one of the mechanisms that the ETS affects the environmental performance of enterprises. We count part of major countries in the inventory of industrial robots in 2001–2019, as shown in Figure 5. China surpassed Japan for the first time in 2016 to become the country with the largest stock of industrial robots in the world. The rapid development of robot applications is bound to have a profound impact on the production mode of enterprises. It can also be seen from the Figure 5 that countries that have established ETS, such as the United States and Germany, are also implementing automated production through the use of industrial robots. Therefore, this paper uses China’s quasi-natural experiment to explore whether the ETS can improve the environmental performance of enterprises by promoting the application of robot production, which can provide references for countries that not only establish ETS but also hope to achieve a higher degree of automation.

### 4.5. Descriptive Statistics

Table 2 shows the descriptive statistical results of the main variables, ln*Sd* is the natural logarithm of the SO_2_ emissions intensity of enterprises. This paper measures the enterprise environmental performance; the standard deviation is 1.232, indicating that there are great differences in environmental performance among enterprises. The minimum values of the first four variables in the table are all 0, indicating that there are enterprises in the sample that do not emit SO_2_ and apply robot production. When studying the impact of SO_2_ ETS, samples need to be divided into pilot areas and non-pilot areas. This paper uses statistical methods to verify whether there are differences between groups and the degree of those differences.

As shown in Table 3, the pilot areas are characterized by high-pollution emissions and low production efficiency, and the overall environmental performance is poor. Moreover, the application of robots in the pilot areas is not as good as in the non-pilot areas, indicating that the application of technology needs to be improved.

## 5. Empirical Analysis

### 5.1. Benchmark Results

The effective implementation of the SO_2_ ETS is key to studying the relationship between policy and corporate environmental performance. Therefore, this paper first tests the effectiveness of SO_2_ ETS, and the regression results are shown in Appendix A Table A1. The results show that after controlling for time and province fixed effect, whether or not to join other control variables, the pilot SO_2_ ETS can significantly reduce SO_2_ emissions, believed that this policy is effective in terms of emissions.

Therefore, we test the impact of SO_2_ ETS on the SO_2_ emissions intensity of enterprises, and the benchmark regression results are shown in Table 4. The results in column (1) show that after controlling for the fixed effects of year, industry, and province, the estimated coefficient of *time × treat* is −0.122 at the 1% significance level, indicating that the SO_2_ ETS can significantly reduce the SO_2_ emissions intensity of enterprises—that is, the SO_2_ ETS can improve the environmental performance of enterprises. The column (2) further adds control variables; the regression results remain the same. Additionally, considering the firm characteristics that do not change with time, we further use the two-way fixed effects model which controls both time and firm effects in columns (3) and (4). The results show that the estimated coefficient of *time × treat* is almost the same with the first two columns.

In conclusion, the above results indicate that the SO_2_ ETS can significantly improve the environmental performance of enterprises—that is, hypothesis 1 is valid. The reason may be that the SO_2_ ETS aims to control the total amount of pollutants discharged by enterprises. On the one hand, it can put pressure on enterprises to discharge pollutants, and then encourage enterprises to take the initiative to reduce the total emission of pollutants through technological progress and other ways. On the other hand, it can also cause certain cost pressures to enterprises, which prompts enterprises to improve production technology and create more profits by improving productivity. Under the dual pressure, the SO_2_ ETS effectively reduces the emissions intensity of SO_2_ and improves the environmental performance of enterprises. Moreover, for enterprises with low emissions, additional income from the sale of surplus emission rights can be used to improve production environment and increase production capacity, which can improve environmental performance. For enterprises with high demand for pollution discharge, compared with reducing the amount of pollution discharge by reducing output to reduce the cost of pollution discharge, enterprises are more inclined to obtain additional revenue by improving productivity to make up for this cost. Therefore, whether high-emissions or low-emissions enterprises, the SO_2_ ETS can improve the environmental performance of them.

### 5.2. Parallel Trend Test

The parallel trend hypothesis is a prerequisite for using the DID model. The parallel trend in this study means that the development trend of the outcome variables in the treatment group and the control group was consistent before the implementation of SO_2_ ETS, but the parallel trend was broken after the implementation of the policy. To ensure that this assumption is met, we draw on the Event Study Approach proposed by Jacobson et al. (1993) [57] to test the dynamic effects of pilot policy and the hypothesis of parallel trend, constructing the following model:

(16) $$\ln Sd_{isct}=\beta_{0}+\sum_{t=2003}^{2013}\beta_{t}treat\times \gamma_{t}+\lambda X+\delta_{i}+\mu_{s}+\eta_{c}+\gamma_{t}+\varepsilon_{isjt}$$

The subscripts *i*, *s*, *c*, and *t* indicate the enterprise, industry, province, and year, respectively. The selection of control variables is consistent with Equation (15).

**Figure 6:**
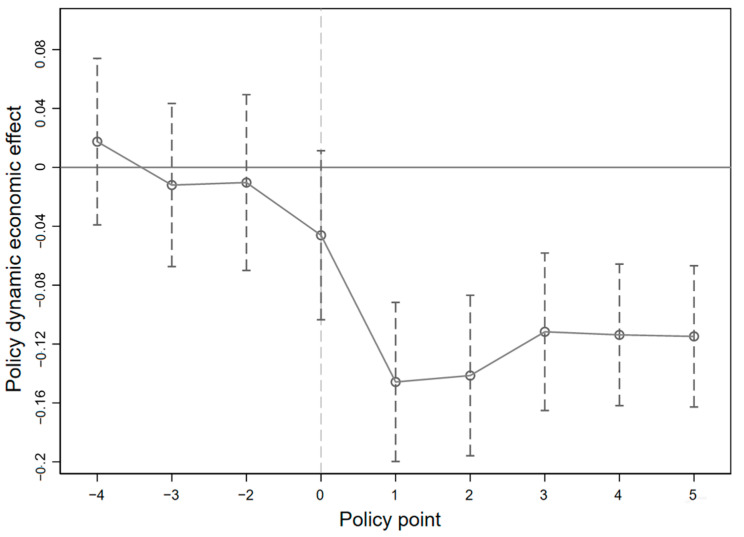
Parallel trend test and dynamic policy effect analysis of OLS.

**Figure 7:**
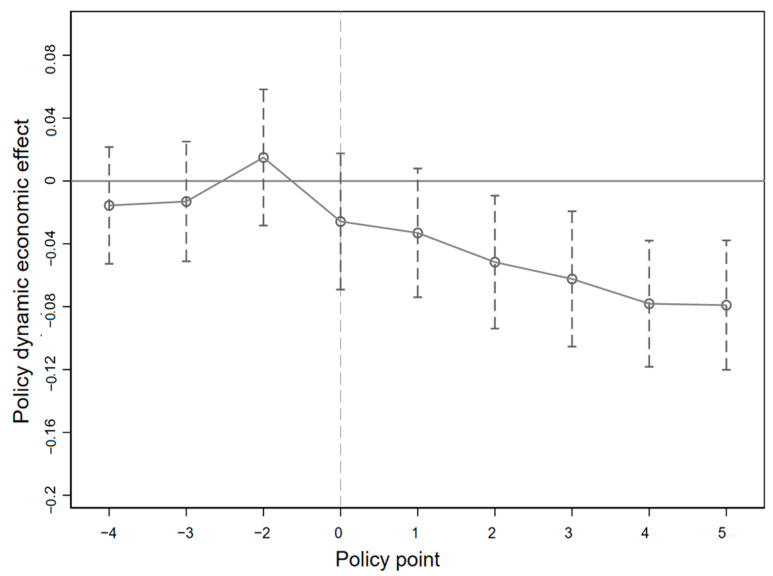
Parallel trend test and dynamic policy effect analysis of FE.

The results of parallel trend test and dynamic policy effect analysis are shown in Table 5 and Figure 6 and Figure 7 plots the estimation results of $\beta_{t}$ under 95% confidence intervals. The Figure 6 controls the characteristics of industries and provinces that do not change over time, and the Figure 7 further controls the characteristics of enterprises that do not change over time. The results of the two figures are basically the same. The regression results in Table 5 show that pilot emissions trading policy launched four years before the corresponding coefficient by significance test, which means that during this period, the variation trend of SO_2_ emission intensity in the treatment group and the control group satisfies the hypothesis of parallel trend. Therefore, the regression coefficients of the core explanatory variable in the benchmark regression can accurately reflect the improvement effect of emission trading policies in pilot areas on enterprise environmental performance.

### 5.3. Robustness Discussion

#### 5.3.1. Screening Sample

To avoid the influence of extreme values on the benchmark regression results, the study samples were censored by 1% and 5% according to the explained variables, and Equation (15) was regressed again. The regression results are shown in Table 6. The results show that the estimated coefficients in OLS and FE regression models both pass the significance test at the level of 1% after the extreme values are removed, which is consistent with the results of the benchmark regression.

#### 5.3.2. Adding Benchmark Variables Alleviates the Impact of Selection

The ideal case for using the DID model is the randomness of the selection of the pilot cities. If the pilot list of emissions trading is related to the city’s economic development status and background factors of emission control, then the difference of these factors may have different effects on enterprises over time, resulting in bias in the regression results. To avoid the influence of non-randomness in the selection of the pilot list, we refer to the methods of Edmonds et al. (2020) [58] and Lu et al. (2017) [59] to add the interaction term between the urban benchmark factors and time linear trend into Equation (15), resulting in the following:

(17) $$\ln Sd_{isct}=\beta_{0}+\beta_{1}time\times treat+\beta_{2}X+\xi Z_{c}\times trend_{t}+\delta_{i}+\mu_{s}+\eta_{c}+\gamma_{t}+\varepsilon_{isjt}$$

where *Z* represents the dummy variable of urban benchmark factors, including whether the city belongs to the “two-control area” or whether it is a provincial capital city, trend is the time trend term.

The “two-control area” refers to the acid rain control areas and sulfur dioxide pollution control areas. Cities with serious acid rain and sulfur dioxide pollution are not included in the sulfur dioxide control zone, but are included in the acid rain control zone. Therefore, the acid rain zone includes cities with high sulfur dioxide emissions, and the “two- control zone” may be used as a reference for the pilot list of SO_2_ ETS. Additionally, the SO_2_ ETS is based on provinces, and provincial capitals are representative cities of provinces. Generally speaking, provincial capitals have higher government supervision and better policy implementation. After adding the interaction term of the urban benchmark variable and time linear trend, the regression results are shown in Table 7. The results show that the SO_2_ ETS still have significantly promoted the improvement of enterprise environmental performance, which is consistent with the benchmark regression results. As a matter of fact, the pilot cities with SO_2_ ETS are located in different geographical locations and scattered throughout China, with different levels of economic development, which to a certain extent is random.

#### 5.3.3. Excluding Special Samples

The existence of special samples will bias the regression results. The DID model is designed to analyze the changes between samples before and after the policy, while some enterprises established after the implementation of the SO_2_ ETS have been affected by this policy in the initial decision making. Therefore, the pilot policy cannot be regarded as a relatively exogenous policy shock, which will cause deviation in the results. To exclude the influence of above special samples, we eliminate the enterprises in the pilot area that were established after the promulgation of the 2007 policy and re-estimate Equation (15). The results are shown in column (1) and (2) of Table 8. Additionally, the emission rights pilot policy imposes emissions reduction pressure and cost pressure on enterprises in pilot areas. To avoid these costs, enterprises have the incentive to flee, and some enterprises may move from pilot areas to non-pilot areas, thus causing bias to the results. In this regard, we collected and sorted the registered address information of enterprises and found that 25 enterprises changed their registered addresses after the implementation of the policy. In this paper, the 25 enterprises that may affect the estimation results are excluded, and the regression results are shown in columns (3) and (4) of Table 8. Results in Table 8 show that after removing the samples that may interfere with the regression results, the regression coefficient of *time × treat* is still negative at the significance level of 1%, indicating that the SO_2_ ETS has a robust effect on the improvement of enterprise environmental performance.

#### 5.3.4. Excluding Other Policy Distractions

During the sample period, other relevant policies may also affect the environmental performance of enterprises in the pilot area, thus causing bias to the benchmark estimation results. Considering that the CO_2_ ETS launched in Beijing, Tianjin, Shanghai, Chongqing, Guangdong, Hubei, and Shenzhen in 2013 may affect the environmental performance of enterprises in the pilot areas, this paper adopts two methods to eliminate the impact of this policy. First, the sample period is changed to 2000–2012 before the full launch of CO_2_ ETS. The regression results are shown in column (1) and (2) of Table 9. Second, the regions that overlap with the CO_2_ ETS (Tianjin, Hubei, and Chongqing) are excluded. The regression results are shown in columns (3) and (4) of Table 9. The results show that after removing the CO_2_ ETS, the regression coefficient of *time × treat* is still significantly negative, and the benchmark regression results of this paper are robust.

In addition, considering China’s simultaneous promotion of “energy saving” and “emission reduction”. While implementing pilot policies on SO_2_ ETS and CO_2_ ETS, the government has also implemented energy policies aimed at improving energy efficiency, which are likely to improve the environmental performance of enterprises. Therefore, when considering the impact of SO_2_ ETS on the environmental performance of enterprises, the impact of energy policies (including energy structure adjustment policies and energy conservation policies) should be excluded. Energy structure adjustment policy addresses traditional energy transformation. Owing to China’s energy production and consumption structure has been dominated by coal for a long time. The implementation of policy object is focused on coal-consuming provinces. Traditional energy transformation can help enterprises to improve energy efficiency and reducing emissions of pollutants. The energy-saving policy was enacted in June 2011. The Ministry of Finance and the National Development and Reform Commission of China have implemented the policy of Comprehensive Demonstration Cities for Fiscal Policies on Energy Conservation and Emission Reduction to achieve energy conservation and emission reduction through financial support. To exclude the influence of energy policies, we remove the provinces with high coal consumption and the comprehensive demonstration cities of energy conservation and emissions reduction finance, re-estimating Equation (15). The results in column (5) and (6) of Table 9 show that after controlling for the impact of energy restructuring policies and energy conservation policies, the conclusion that SO_2_ ETS has a positive effect on promoting enterprise environmental performance is robust.

#### 5.3.5. PSM-DID

The DID model is used because the experimental group and the control group have homogeneity before the implementation of the policy. However, this assumption is difficult to meet in reality. Therefore, we adopt the Propensity Score Matching (PSM) method to conduct the robustness test. Specific ideas are as follows: the Logit model is adopted, *time × treat* is the dependent variable, and variables such as enterprise age, enterprise size, enterprise ownership, operating profit rate, asset-liability ratio, capital intensity, regional economic structure, and environmental regulation intensity are used as corresponding covariates. The Kernel matching method and Radius matching method are used for sample matching. Therefore, the balance test results after matching are obtained in this paper. As can be seen from the test results in Appendix A Table A2 and Table A3, the absolute value of standard deviations of all matched variables except environmental regulation intensity (*Regulation*) after matching are less than 10%, which indicated that there is no significant difference in the mean values of matched variables between the treatment group and the control group after matching. Therefore, matching meets the balance test. On the basis of PSM, a new control group with similar characteristics to the treatment group was obtained. The DID model is used to investigate the impact of SO_2_ ETS on environmental performance. The results in Table 10 show that no matter which matching method is adopted, the coefficient of *time × treat* reaches the significance level of 1%. Therefore, the benchmark regression results in this paper pass the robustness test of PSM-DID.

#### 5.3.6. Placebo Test

**Figure 8:**
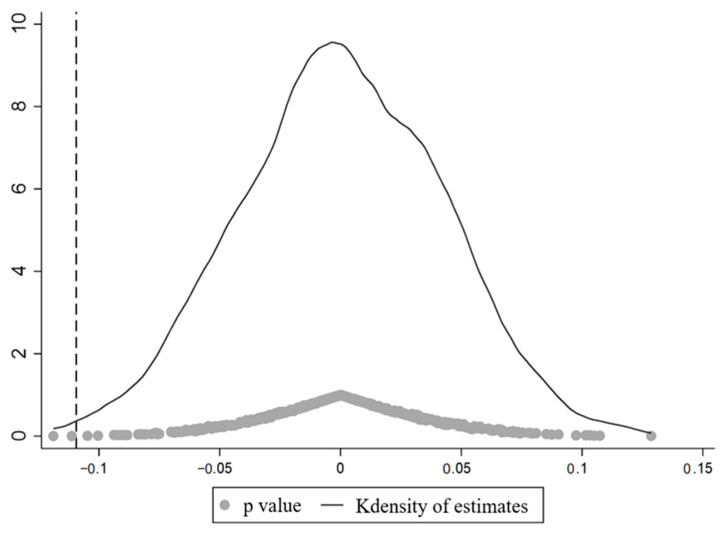
Results of the placebo test of OLS.

**Figure 9:**
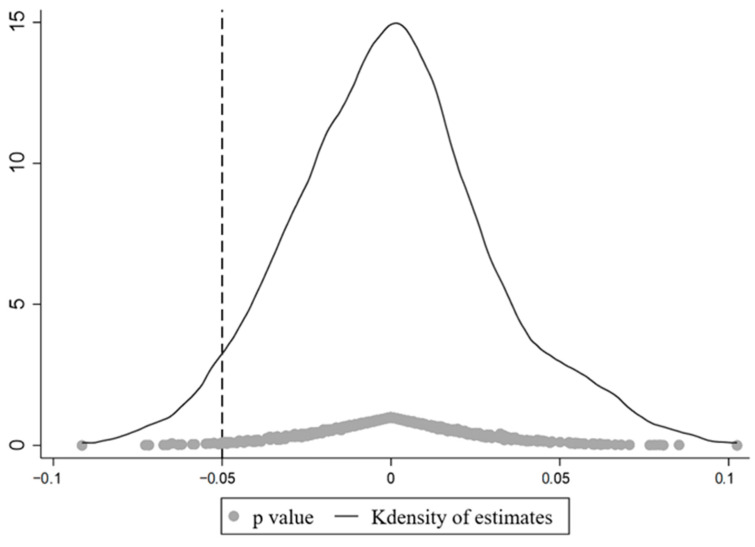
Results of the placebo test of FE.

To further test whether the results of this paper are caused by other unobservable random factors, we conduct a placebo test by randomly generating pilot provinces according to Cai et al. (2016) [60]. The specific operation is as follows: 11 provinces are randomly selected from the 30 pilot provinces as the treatment group, assuming that the selected provinces are the pilot areas of the SO_2_ ETS, and the unselected areas are the control group. This process is repeated 500 times to obtain 500 regression estimation coefficients. Figure 8 and Figure 9 reports the distribution of estimated coefficients and *p*-values for 500 random samples. The estimated coefficients are clustered around the zero point, with *p*-values mostly greater than 0.1, and the previous estimates are significant outliers in the placebo test. The relevant regression results show that the benchmark regression results pass the placebo test.

#### 5.3.7. Test the Endogeneity

The pilot areas of SO_2_ ETS are widely distributed and do not have similar economic development levels and trends, which seems to indicate that the selection of pilot areas is random. However, it cannot be not excluded that some potentially unobservable factors affect the selection of pilot areas. Therefore, this paper adopts appropriate instrumental variables and uses the two-stage least squares (2sls) method to alleviate possible policy endogeneity problems. Referring to the practice of Hering and Poncet (2014) [36], we select the ventilation coefficient of each region as the instrumental variable for whether to be included in the pilot. The ventilation coefficient is selected because the ventilation coefficient is defined as the product of the wind speed and the height of the mixing layer. The wind speed and the height of the mixing layer contribute to the diffusion of pollutants from horizontal and vertical directions and they jointly determine the diffusion speed of pollutants (Jacobson, 2002 [57]). The faster the pollutant spreads and the less sulfur dioxide emissions are monitored, the less likely the area is to be selected as a pilot site. Therefore, the ventilation coefficient is negatively correlated with the expected probability of the included pilot area, and this instrumental variable meets the requirement of correlation. Additionally, the ventilation coefficient is determined by meteorological systems and geographical conditions, which satisfies the exogeneity condition of instrumental variables.

The ventilation coefficient is obtained from the ECMWF Re-Analysis database of the European Centre for Intermediate Weather Forecasts, and the product of wind speed information at a height of 10 m and boundary layer height is used as the ventilation coefficient. Because the SO_2_ ETS was officially launched in 2007, the average ventilation coefficient before the first 5 years of the policy (namely, 2003–2007) is taken as the instrumental variable. Considering that the SO_2_ ETS is implemented by provinces, the average ventilation coefficients at the province level (*tf_s*) and the city level (*tf_c*) are selected as instrumental variables. The results in Table 11 show that the impact of the SO_2_ ETS on emissions intensity is still significantly negative, indicating that the pilot policy can significantly improve the environmental performance of enterprises and that this result is not caused by sample selection bias.

## 6. Impact Mechanisms of the Channel

The above research results show that the SO_2_ ETS can improve the environmental performance of enterprises. The next question to explore involves what are the internal transmission mechanisms. As shown in Figure 1 of the theoretical analysis section, SO_2_ ETS can not only directly improve the environmental performance of enterprises by reducing the total amount of emissions, but also improve the environmental performance by encouraging enterprises to apply robot production and improve the total factor productivity.

### 6.1. Channel 1: Pollutant Discharge

Reducing emissions is the most direct way to improve the environmental performance of enterprises, and it is also the constraint means of the SO_2_ ETS. Therefore, it is necessary to examine the impact of SO_2_ ETS on the total emissions of enterprises. To test whether this mechanism is valid, the explained variable in Equation (15) is replaced by enterprise sulfur dioxide emissions (ln*SO*_2_), with other variables remaining unchanged. Table 12 reports the corresponding regression results. The results show that whether or not other influencing factors are controlled, the regression coefficients of *time × treat* are negative at the significance level of 1%, indicating that the policy can significantly reduce the total amount of pollutants emitted by enterprises. Therefore, it is effectively verified that the SO_2_ ETS affects the environmental performance of enterprises by way of pollutant discharge. Therefore, Hypothesis 2 is valid.

### 6.2. Channel 2: Robot Application and Total Factor Productivity

To test whether the SO_2_ ETS can promote the improvement of total factor productivity so as to improve the environmental performance of enterprises by promoting the application of robot production, two regressions are conducted in this paper. One is the study on the impact of the SO_2_ ETS on the application of robots in enterprises; the other is the effect of robot application on the total factor productivity of enterprises.

To study the relationship between the SO_2_ ETS and the application of robots, the amount of robots (*RobotV*) and the number of robots (*RobotN*) in the customs database are selected as proxy variables. We adopt two methods for verification: (1) The two-way fixed effect model is used to control the firm, time, industry, and province fixed effects. The regression results are shown in column (1) and (2) of Table 13. The regression coefficients of *time × treat* are positive at the significance level of 5% to 10%, indicating that the SO_2_ ETS significantly promotes the application of robots in enterprises. (2) Considering that there are a large number of zeros in the robot data in our sample, sample selectivity bias may occur if only a two-way fixed-effect model regression is considered. However, the Heckman two-stage model is suitable for solving the endogeneity problem caused by sample selection bias. Therefore, the Heckman two-stage model is also selected for regression analysis in this paper. The specific ideas are as follows: First, the Probit estimation of the enterprise robot in the first stage is conducted, and the ventilation coefficient (*tf*) in the endogeneity test is added as the exogenous control variable to calculate the inverse Mills ratio (*lambda*). Take *lambda* as the control variable; it is then included in the estimation equation of the application of enterprise robots by the emissions trading pilot policy for regression analysis. The regression results are shown in columns (3) and (4) of Table 13. The results still show that the SO_2_ ETS can significantly improve the application of enterprise robots.

To study the relationship between robot application and enterprise total factor productivity, the semi-parametric method proposed by Olley and Pakes (1996) [52] and Levinsohn and Petrin (2003) [53] is used to measure the enterprise total factor productivity as the explained variable. The regression model controlling the firm fixed effect can effectively control the missing variables that do not change with time at the firm level. However, when the variation degree of the core explanatory variables is local, the research model controlling the firm fixed effect is prone to research bias (de Haan, 2021 [61]). Considering that the core explanatory variable used is robots, and that changes in the number or value of imported robots only occur in enterprises that use robots for production, it can be regarded that the variation degree of robots is local. Therefore, controlling the fixed effect of the firm will result in biased results. Columns (1) and (2) of Table 14 show the regression results of robot application on firm TFP when controlling for time, province, and industry fixed effects. It can be seen that robot production can significantly improve the total factor productivity of enterprises.

Additionally, to alleviate the endogeneity problem of robot application, we further select the industrial producer purchase price index as the instrumental variable of robot regression. The purchasing price index of industrial producers can reflect the changing trend and degree of the purchasing price of intermediate product input and the changing of enterprise costs when the resource input is unchanged. Robots can replace workers in production but cannot replace resource input. When the input cost of intermediate product rises, enterprises do not have enough funds to import robots. Therefore, it is believed that there is a negative correlation between the purchasing price index of industrial producers and robots. Moreover, the values of the insufficient identification test and the weak identification test statistics are both large, which rejects the hypothesis of insufficient identification and weak identification, indicating that the instrumental variables selected in this paper are related to the robots. The purchasing price index of industrial producers is collected by the state, which satisfies the condition of exogeneity of instrumental variables.

Columns (3) and (4) of Table 14 show the regression results of the two-stage least squares method, and the results still show that the application of robot production has a significant effect on promoting the total factor productivity of enterprises.

In summary, this study finds that the pilot emissions trading policy can promote enterprises to import robots, and the application of robots in production can significantly promote the total factor productivity of enterprises, thus improving the environmental performance of enterprises. Hypothesis 3 is valid.

## 7. Further Analysis

All three hypotheses concerning the impact of SO_2_ ETS on corporate environmental performance have been verified above, and the following supplementary discussions will be made from the perspective of regional heterogeneity.

Enterprises discharge pollutants largely because of the input of energy materials in production. As an important strategic resource, energy utilization efficiency is affected by the dynamic of resource endowment. Therefore, the use of energy by enterprises is closely related to a region’s resource situation. China’s Circular on the Issuance of the National Plan for Sustainable Development of Resource-based Cities has identified 262 resource-based cities, county-level cities, or municipal districts, which are classified into four types according to their resource affluence—growth, maturity, decline, and regeneration. According to this division method, this paper conducts group regression to test whether emissions trading pilot policies have heterogeneous effects on different types of resource-based cities. The results in column (1) of Table 15 show that the SO_2_ ETS can significantly improve the environmental performance of enterprises in resource-based cities. Columns (2)–(4) analyze the impact of SO_2_ ETS on the heterogeneity of different types of resource-based cities—among them, the effect of SO_2_ ETS on improving the environmental performance of enterprises in declining resource cities is significant at the 1% level, the effect on mature and regenerative resource cities is not significant, while it has a restraining effect on the environmental performance of enterprises in growing resource cities.

The reason for this result may be that the resources of declining resource-based cities are decreasing day by day and that the energy costs of enterprises are gradually increasing. Owing to the objective constraint of profit maximization, most enterprises pay attention to saving energy consumption or strengthening R&D investment in energy utilization technology. The SO_2_ ETS has encouraged enterprises to reduce emissions and generate more revenue from the sale of emissions permits so that more funds can be used to improve energy efficiency. Therefore, for enterprises in declining resource-based cities, the SO_2_ ETS can significantly improve their environmental performance. Growing resource-based cities have the richest resources and the lowest energy costs. Enterprises with high or low energy utilization efficiency in these cities are able to exploit and use more energy, so they have a weak motivation to improve energy utilization efficiency. Moreover, since low-cost energy input can obtain more profits, which is enough to offset the cost of purchasing emission rights, enterprises prefer to purchase emission rights rather than improve energy utilization efficiency. Therefore, SO_2_ ETS have a restraining effect on the environmental performance of enterprises in growing resource-based cities. Additionally, the resource richness and energy cost of mature resource-based cities are at a medium level. The benefit for an enterprise investing more energy is not enough to make up for purchasing additional energy and the cost of emissions. The enterprise is not necessary to reduce energy input to save emissions for extra income, so the enthusiasm of enterprises to participate in the emissions trading is weak. The SO_2_ ETS has no significant effect on the environmental performance of enterprises in mature resource-based cities. Regenerative resource-based cities are relatively short of resources, and the pressure faced by enterprises primarily comes from energy exhaustion rather than emissions, which makes the effect of SO_2_ ETS itself insignificant.

## 8. Conclusions 

Rapid economic development has brought about the globalization of emissions and other forms of pollution. Green production is the common goal of all countries. The main purpose of environmental regulation is to control the total emissions of pollutants. If the environmental regulation policy can achieve the emissions reduction effect as scheduled, it would also improve the environmental performance of enterprises, thus achieving a “win-win” situation at the national and enterprise levels. Based on the heterogeneous enterprise model developed by Melitz (2003) [20], this paper constructs the emissions trading market, introduces the production choice of robot application by enterprises, and investigates the impact of SO_2_ ETS on enterprises’ environmental performance and its internal mechanism. Furthermore, this paper used the matching data of the China Industrial Enterprise Database, the China Industrial Pollution Source Key Investigation Enterprise Database, and the Customs Database from 2000 to 2013, and China’s 2007 SO_2_ ETS as a quasi-natural experiment. The identification framework of the DID method was constructed to control potential endogeneity problems. This paper empirically examines the effects and mechanisms of SO_2_ ETS on corporate environmental performance. The results show that SO_2_ ETS significantly reduces the emissions intensity of sulfur dioxide so as to improve the environmental performance of enterprises. This conclusion has passed a series of robustness tests, such as the parallel trend test and the PSM-DID test. Additionally, we found that enterprises improve their environmental performance through the reduction of emissions and the application of robot production to improve total factor productivity. Heterogeneity analysis shows that declining-resource and growing-resource cities are more sensitive to the SO_2_ ETS, but the SO_2_ ETS is only beneficial to the environmental performance of declining-resource cities and has a restraining effect on the environmental performance of growing-resource cities. 

According to the research results of this paper, some policy suggestions are put forward. First of all, to China: (1) Give full play to the decisive role of the market mechanism and give more recognition to the ETS. China’s environmental regulation mode is changing from the traditional command-control mode to the market-incentive mode. The empirical results of this paper prove that the pilot ETS can not only effectively reduce the pollution emissions in pilot areas but can also improve the environmental performance of enterprises so as to achieve a win-win situation at the national and enterprise levels. The study fully affirms the market-based environmental regulation represented by SO_2_ ETS and insists on giving full play to the decisive factor of the market in resource allocation. Additionally, the success of China’s 2007 SO_2_ ETS, as an early implementation of market-based environmental rights trading policy, provides a reference and inspiration for the Chinese government to comprehensively promote carbon emission rights and improve water rights, energy rights, and other trading plans in the future. (2) Attach importance to the promotion effect of environmental regulation on robot application and the employment substitution effect of robots. This paper argues that SO_2_ ETS increases the number and amount of robots, the use of robots in production can significantly improve the total factor productivity of enterprises. Therefore, the application of robots can result in an outstanding performance with respect to emissions reduction and efficiency improvement. A large number of applications of robots will be an ongoing trend. However, the existing literature shows that the application of robots will reduce the labor demand and wages in the equilibrium, which poses a threat to workers. Therefore, the government should consider how to encourage enterprises to use robot production to reduce pollution emissions while avoiding the substitution of robots for jobs. (3) The availability of regional resources affects the effective implementation of SO_2_ ETS. The results of the hetereogeneity analysis show that in regions with relatively rich resources, the coal price is low; thus, SO_2_ ETS cannot produce effects. When resources are easily available, SO_2_ ETS even encourages enterprises to purchase additional emission rights to emit more pollution, which has a negative impact on environmental performance. By contrast, in regions with relatively low resources, the coal price is high, and SO_2_ ETS can effectively improve the environmental performance of enterprises. However, when regional resources are completely unavailable, the policy effectiveness disappears. Therefore, the government should control regional coal prices and maintain coal constraints for growing resource-based cities, for example, by setting minimum coal prices. Other environmental regulation means should be implemented for regenerative cities, such as innovation incentives for enterprises in the region.

Second, there are policy recommendations for countries that have established ETS, such as the United States and some European countries that have a large demand for robots: (1) More attention should be paid to the emission intensity of pollutants rather than the total amount. The purpose of ETS is to reduce the total amount of pollutant emissions. Some enterprises may reduce the amount of pollutant emissions by reducing production, which can protect the environment but is not conducive to the profits of enterprises. This paper finds that the robot application can avoid the above situation. Robots can improve the production efficiency of enterprises, that is, improve the efficiency of energy utilization, and the improvement of production efficiency helps to reduce the emission intensity of pollutants. The government should encourage enterprises to adopt greener production equipment and realize intelligent and green production at the same time. (2) Targeted support policies should be administered to help enterprises solve the problem of not being able to use robots. For example, ETS can encourage companies to use robots for production, but due to financial constraints, companies may not be able to pay for robots. In this case, the government needs to provide financial support. For example, the government can formulate a kind of R&D tax deduction policy to promote enterprises to apply robot production. In addition, the government should also investigate other difficulties hindering the use of robots by enterprises and formulate targeted policies so that more enterprises can realize green production transformation.

Finally, there are some suggestions for countries without ETS. After the implementation of ETS in developed countries, China can learn from their experience in the implementation process, and combine it with its own unique system advantages, which may produce different effects from the developed countries. China’s EST pilot policy provides cases of developing countries and shows that market based environmental regulation represented by ETS is worth promoting and learning. Countries should actively establish ETS to reduce pollutant emissions by taking advantage of robot production. In addition, the experience of SO_2_ ETS can be used to solve the problem of CO_2_ emissions to mitigate global warming. We believe that with the joint efforts of all countries, the UN 2030 Sustainable Development Goals will be achieved.

## Figures and Tables

**Table 1:** Definition and measurement of variables.

Variable	Variable Definitions	Method of Measurement
*Sd*	Environmental performance	SO_2_ emission intensity, =SO_2_ emissions/total industrial output
*treat*	Policy variable	Whether the enterprise is located in the pilot area of SO_2_ EST—if the value is 1, and 0 otherwise.
*time*	Time variable	Whether the sample time is after 2007—if the value is 1, and 0 otherwise.
*SO*_2_	SO_2_ emissions	Total amount of SO_2_ produced by enterprises
*RobotV*	Robot application	The amount of importing robots, unit ten thousand yuan.
*RobotN*	Robot application	The number of importing robots.
*Tfp_op*	Total factor productivity (op)	Based on the method of Olley and Pakes (1996) [52].
*Tfp_lp*	Total factor productivity (lp)	Based on the method of Levinsohn and Petrin (2003) [53].
*Age*	Enterprise age	=Year of sample–Year of establishment
*Scale*	The enterprise scale	=ln (Employees of enterprises + 1)
*Owner*	Ownership of enterprise	Whether the enterprise is a state-owned enterprise—if the value is 1; and 0 otherwise.
*Kl*	Capital intensity	=Total assets/Total operating income
*Alr*	Asset-liability ratio	=Total liabilities/Total assets
*Se_gdp*	Regional economic structure	=The proportion of tertiary industry in GDP/The proportion of secondary industry in GDP
*Regulation*	Environmental regulation intensity	=Total revenue from sewage charge/Number of units paying pollution charges

**Table 2:** Descriptive statistics.

Variable	N	Mean	S.D.	Min	Max
ln*Sd*	103,651	1.128	1.232	0.000	14.067
ln*SO*_2_	103,651	7.746	4.248	0.000	18.500
ln*RobotV*	138,023	0.044	0.729	0.000	16.584
ln*RobotN*	138,023	0.006	0.126	0.000	10.309
*Tfp_op*	131,840	3.799	0.933	0.000	8.822
*Tfp_lp*	131,840	6.812	1.112	1.945	11.560
*Age*	138,023	1.416	1.223	0.000	15.600
*Scale*	138,023	6.040	1.165	2.197	12.316
*Owner*	138,023	0.047	0.211	0.000	1.000
*Kl*	138,023	0.011	0.285	0.000	78.066
*Alr*	138,023	0.591	6.326	0.000	1779.417
*Se_gdp*	135,541	0.509	0.767	0.160	0.900
*Regulation*	138,023	3.351	2.655	0.163	27.467

**Table 3:** Comparison between the treatment and the control group.

		Treatment Group		Control Group		Differences	
		N	Mean	N	Mean	Mean	*t*-Value
Dependent & Independentvariable	ln*Sd*	44,911	8.136	58,740	7.449	0.687 ***	25.877
	ln*SO*_2_	44,911	1.184	58,740	1.085	0.099 ***	12.784
	*Tfp_op*	57,947	6.762	73,893	6.852	−0.090 ***	−14.650
	*Tfp_lp*	57,947	3.747	73,893	3.839	−0.093 ***	−17.901
	ln*RobotV*	60,321	0.034	77,702	0.053	−0.018 ***	−4.658
	ln*RobotN*	60,321	0.005	77,702	0.008	−0.003 ***	−4.324
Firm-level variables	*Age*	60,321	1.453	77,702	1.387	0.066 ***	9.923
	*Scale*	60,321	0.050	77,702	0.045	0.005 ***	4.202
	*Owner*	60,321	6.004	77,702	6.068	−0.065 ***	−10.248
	*Kl*	60,321	0.010	77,702	0.012	−0.001	−0.804
	*Alr*	60,321	0.620	77,702	0.568	0.052	1.507
	*Se_gdp*	59,632	0.527	75,909	0.494	0.032 ***	78.818
	*Regulation*	60,321	3.849	77,702	2.964	0.885 ***	62.282

Significance: *** 1%.

**Table 4:** Benchmark Results.

Dep. Var.	OLS		FE	
	(1)	(2)	(3)	(4)
*time × treat*	−0.122 ***	−0.112 ***	−0.061 ***	−0.051 ***
	(0.014)	(0.014)	(0.014)	(0.014)
*Age*		0.026 ***		0.012 **
		(0.003)		(0.006)
*Owner*		0.167 ***		0.074 **
		(0.019)		(0.031)
*Scale*		−0.052 ***		0.001
		(0.003)		(0.006)
*Kl*		−0.007		−0.013 **
		(0.014)		(0.007)
*Alr*		0.002 ***		0.001 ***
		(0.001)		(0.000)
*Se_gdp*		0.029		0.112
		(0.056)		(0.098)
*Regulation*		−0.017 ***		−0.012 ***
		(0.003)		(0.003)
_cons	1.154 ***	1.440 ***	1.126 ***	1.063 ***
	(0.004)	(0.035)	(0.004)	(0.065)
Control variables	×	√	×	√
Year FE	√	√	√	√
Sector FE	√	√	√	√
Province FE	√	√	√	√
Firm FE	×	×	√	√
Observations	103,650	101,417	93,391	91,475
R-squared	0.225	0.227	0.795	0.793

Notes: Table 4 reports the results of the relationship between SO_2_ ETS and a firm’s environmental performance. Using the fixed effect model, the Hausman test result is 0.0277. Significance: *** 1%, ** 5%.

**Table 5:** Parallel trend test and dynamic policy effect analysis.

	OLS	FE
*Pre4*	0.017	−0.016
	(0.029)	(0.019)
*Pre3*	−0.012	−0.013
	(0.028)	(0.019)
*Pre2*	−0.010	0.015
	(0.030)	(0.022)
*current*	−0.046	−0.026
	(0.029)	(0.022)
*Post1*	−0.146 ***	−0.033
	(0.028)	(0.021)
*Post2*	−0.141 ***	−0.052 **
	(0.028)	(0.022)
*Post3*	−0.112 ***	−0.062 ***
	(0.027)	(0.022)
*Post4*	−0.114 ***	−0.078 ***
	(0.025)	(0.020)
*Post5*	−0.115 ***	−0.079 ***
	(0.024)	(0.021)
Control variables	√	√
Year FE	√	√
Sector FE	√	√
Province FE	√	√
Firm FE	×	√
Observations	103,650	101,417
R-squared	0.225	0.227

Note: Robust Standard errors in parentheses; *** 1%, ** 5%.

**Table 6:** Screening sample.

Dep. Var.	Truncation 1%		Truncation 5%	
	(1)	(2)	(3)	(4)
*time × treat*	−0.118 ***	−0.060 ***	−0.117 ***	−0.047 ***
	(0.013)	(0.013)	(0.011)	(0.011)
*Age*	0.027 ***	0.013 **	0.027 ***	0.017 ***
	(0.003)	(0.005)	(0.003)	(0.005)
*Owner*	0.156 ***	0.080 ***	0.131 ***	0.058 **
	(0.017)	(0.029)	(0.015)	(0.026)
*Scale*	−0.055 ***	−0.002	−0.050 ***	−0.003
	(0.003)	(0.006)	(0.003)	(0.005)
*Kl*	−0.004	−0.004	0.001	0.001
	(0.014)	(0.004)	(0.012)	(0.002)
*Alr*	0.002 ***	0.001 ***	0.002 ***	0.001 ***
	(0.000)	(0.000)	(0.000)	(0.000)
*Se_gdp*	0.099 *	0.127	0.146 ***	0.125
	(0.052)	(0.089)	(0.045)	(0.080)
*Regulation*	−0.016 ***	−0.011 ***	−0.012 ***	−0.009 ***
	(0.002)	(0.002)	(0.002)	(0.002)
_cons	1.154 ***	1.440 ***	1.126 ***	1.063 ***
	(0.004)	(0.035)	(0.004)	(0.065)
Year FE	√	√	√	√
Sector FE	√	√	√	√
Province FE	√	√	√	√
Firm FE	×	√	×	√
Observations	103,650	101,417	93,391	91,475
R-squared	0.225	0.227	0.795	0.793

Note: Robust Standard errors in parentheses; *** 1%, ** 5%, * 10%.

**Table 7:** Adding benchmark variables alleviates the impact of selection.

Dep. Var.	Dual Control Area		Provincial Capital	
	(1)	(2)	(3)	(4)
*time × treat*	−0.115 ***	−0.050 ***	−0.125 ***	−0.058 ***
	(0.014)	(0.014)	(0.015)	(0.015)
*Age*	0.026 ***	0.013 **	0.026 ***	0.012 **
	(0.003)	(0.006)	(0.003)	(0.006)
*Owner*	0.168 ***	0.075 **	0.167 ***	0.075 **
	(0.019)	(0.031)	(0.019)	(0.031)
*Scale*	−0.051 ***	0.001	−0.051 ***	0.001
	(0.003)	(0.006)	(0.003)	(0.006)
*Kl*	−0.007	−0.013 **	−0.007	−0.013 **
	(0.014)	(0.007)	(0.014)	(0.007)
*Alr*	0.002 ***	0.001 ***	0.002 ***	0.001 ***
	(0.001)	(0.000)	(0.001)	(0.000)
*Se_gdp*	0.043	0.113	−0.036	0.093
	(0.056)	(0.100)	(0.057)	(0.099)
*Regulation*	−0.019 ***	−0.012 ***	−0.017 ***	−0.012 ***
	(0.003)	(0.003)	(0.003)	(0.003)
_cons	1.492 ***	0.536	1.348 ***	0.879 ***
	(0.055)	(0.327)	(0.062)	(0.079)
Year FE	√	√	√	√
Sector FE	√	√	√	√
Province FE	√	√	√	√
Firm FE	×	√	×	√
Observations	101,706	91,761	101,706	91,761
R-squared	0.227	0.793	0.228	0.793

Note: Robust Standard errors in parentheses; *** 1%, ** 5%.

**Table 8:** Excluding special samples.

Dep. Var.	Enterprises Established after the Implementation of the Policy		Enterprises that Fled after the Policy Was Implemented	
	(1)	(2)	(3)	(4)
*time × treat*	−0.113 ***	−0.050 ***	−0.112 ***	−0.051 ***
	(0.014)	(0.014)	(0.014)	(0.014)
*Age*	0.026 ***	0.012 **	0.026 ***	0.013 **
	(0.003)	(0.006)	(0.003)	(0.006)
*Owner*	0.167 ***	0.073 **	0.166 ***	0.074 **
	(0.019)	(0.031)	(0.019)	(0.031)
*Scale*	−0.052 ***	0.001	−0.052 ***	0.001
	(0.003)	(0.006)	(0.003)	(0.006)
*Kl*	−0.006	−0.013 **	−0.007	−0.013 **
	(0.014)	(0.007)	(0.014)	(0.007)
*Alr*	0.002 ***	0.001 ***	0.002 ***	0.001 ***
	(0.001)	(0.000)	(0.001)	(0.000)
*Se_gdp*	0.039	0.112	0.029	0.113
	(0.056)	(0.098)	(0.056)	(0.098)
*Regulation*	−0.018 ***	−0.012 ***	−0.017 ***	−0.012 ***
	(0.003)	(0.003)	(0.003)	(0.003)
_cons	1.437 ***	1.062 ***	1.440 ***	1.062 ***
	(0.035)	(0.065)	(0.035)	(0.065)
Year FE	√	√	√	√
Sector FE	√	√	√	√
Province FE	√	√	√	√
Firm FE	×	√	×	√
Observations	100,888	91,106	101,375	91,435
R-squared	0.227	0.793	0.227	0.793

Note: Robust Standard errors in parentheses; *** 1%, ** 5%.

**Table 9:** Excluding Other Policy Distractions.

Dep. Var.	Rule Out Carbon Emission Trading Policy in 2013				Rule Out Energy Policy	
	(1)	(2)	(3)	(4)	(5)	(6)
*time × treat*	−0.104 ***	−0.043 ***	−0.137 ***	−0.062 ***	−0.079 ***	−0.057 **
	(0.015)	(0.015)	(0.014)	(0.015)	(0.022)	(0.023)
*Age*	0.027 ***	0.009	0.024 ***	0.009	0.031 ***	0.027 **
	(0.003)	(0.006)	(0.003)	(0.006)	(0.006)	(0.011)
*Owner*	0.159 ***	0.086 ***	0.169 ***	0.089 ***	0.156 ***	0.102 *
	(0.019)	(0.033)	(0.020)	(0.033)	(0.037)	(0.062)
*Scale*	−0.048 ***	−0.002	−0.055 ***	−0.001	−0.034 ***	−0.008
	(0.003)	(0.007)	(0.003)	(0.007)	(0.005)	(0.010)
*Kl*	−0.004	−0.013 **	−0.005	−0.014 **	−0.117	−0.026
	(0.012)	(0.007)	(0.013)	(0.007)	(0.082)	(0.022)
*Alr*	0.002 ***	0.001 ***	0.002 ***	0.001 ***	0.001 ***	0.001 ***
	(0.000)	(0.000)	(0.001)	(0.000)	(0.000)	(0.000)
*Se_gdp*	−0.013	0.226 **	0.030	0.059	−0.632 ***	−0.268 *
	(0.060)	(0.108)	(0.057)	(0.104)	(0.082)	(0.162)
*Regulation*	−0.023 ***	−0.018 ***	−0.019 ***	−0.014 ***	−0.030 ***	−0.022 ***
	(0.003)	(0.003)	(0.003)	(0.003)	(0.007)	(0.007)
Year FE	√	√	√	√	√	√
Sector FE	√	√	√	√	√	√
Province FE	√	√	√	√	√	√
Firm FE	×	√	×	√	×	√
Observations	92,095	81,564	94,663	85,350	42,424	37,748
R-squared	0.232	0.792	0.231	0.792	0.262	0.791

Note: Robust Standard errors in parentheses; *** 1%, ** 5%, * 10%.

**Table 10:** PSM-DID.

Dep. Var.	Kernel Matching		Radius Matching	
	(1)	(2)	(3)	(4)
*time × treat*	−0.112 ***	−0.051 ***	−0.113 ***	−0.050 ***
	(0.014)	(0.014)	(0.014)	(0.014)
*Age*	0.026 ***	0.012 **	0.027 ***	0.012 **
	(0.003)	(0.006)	(0.003)	(0.006)
*Owner*	0.168 ***	0.074 **	0.173 ***	0.074 **
	(0.019)	(0.031)	(0.019)	(0.031)
*Scale*	−0.051 ***	0.001	−0.055 ***	−0.000
	(0.003)	(0.006)	(0.003)	(0.007)
*Kl*	−0.007	−0.013 **	−0.475 ***	−0.038
	(0.014)	(0.007)	(0.056)	(0.047)
*Alr*	0.002 ***	0.001 ***	0.002 ***	0.001 ***
	(0.001)	(0.000)	(0.001)	(0.000)
*Se_gdp*	0.031	0.107	0.041	0.108
	(0.056)	(0.098)	(0.056)	(0.099)
*Regulation*	−0.017 ***	−0.012 ***	−0.017 ***	−0.012 ***
	(0.003)	(0.003)	(0.003)	(0.003)
_cons	1.438 ***	1.066 ***	1.460 ***	1.071 ***
	(0.035)	(0.065)	(0.035)	(0.066)
Year FE	√	√	√	√
Sector FE	√	√	√	√
Province FE	√	√	√	√
Firm FE	×	√	×	√
Observations	101,706	91,761	101,463	91,528
R-squared	0.227	0.793	0.228	0.793

Note: Robust Standard errors in parentheses; *** 1%, ** 5%.

**Table 11:** Test for endogeneity.

Dep. Var.	(1)		(2)	
	*time × treat*	ln*Sd*	*time × treat*	ln*Sd*
*time × tf_s*	−0.023 ***			
	(0.001)			
*time × tf_c*			−0.014 ***	
			(0.001)	
*time × treat*		−0.296 **		−0.280 **
		(0.089)		(0.109)
*Age*	−0.009 ***	0.010 *	−0.009 ***	0.010 *
	(0.002)	(0.006)	(0.002)	(0.006)
*Owner*	−0.002	0.074 **	−0.002	0.074 **
	(0.009)	(0.031)	(0.009)	(0.031)
*Scale*	0.012 ***	0.004	0.012 ***	0.003
	(0.002)	(0.006)	(0.002)	(0.006)
*Kl*	−0.003 **	−0.014 **	−0.002 **	−0.014 **
	(0.001)	(0.007)	(0.001)	(0.007)
*Alr*	−0.000 *	0.001 ***	−0.000 *	0.001 ***
	(0.000)	(0.000)	(0.000)	(0.000)
*Se_gdp*	−0.615 ***	−0.004	−0.661 ***	0.004
	(0.033)	(0.108)	(0.034)	(0.111)
*Regulation*	0.021 ***	−0.008 **	0.020 ***	−0.008 **
	(0.001)	(0.003)	(0.001)	(0.003)
Year FE	√	√	√	√
Sector FE	√	√	√	√
Province FE	√	√	√	√
Firm FE	√	√	√	√
Observations	91,761	91,761	91,761	91,761
R-squared		0.014		0.015

Note: Robust Standard errors in parentheses; *** 1%, ** 5%, * 10%.

**Table 12:** Mechanism test of the influence of the SO_2_ ETS on emissions.

Dep. Var.	OLS		FE	
	(1)	(2)	(3)	(4)
*time × treat*	−0.933 ***	−0.790 ***	−0.229 ***	−0.226 ***
	(0.046)	(0.046)	(0.042)	(0.043)
*Age*		0.161 ***		0.081 ***
		(0.011)		(0.019)
*Owner*		0.516 ***		0.103
		(0.064)		(0.091)
*Scale*		0.720 ***		0.271 ***
		(0.011)		(0.020)
*Kl*		0.226 ***		0.046 ***
		(0.068)		(0.015)
*Alr*		0.006 **		−0.000
		(0.003)		(0.000)
*Se_gdp*		0.568 ***		1.228 ***
		(0.177)		(0.295)
*Regulation*		−0.054 ***		−0.020 ***
		(0.008)		(0.007)
_cons	7.947 ***	3.126 ***	7.875 ***	5.494 ***
	(0.015)	(0.114)	(0.011)	(0.197)
Control variables	×	√	×	√
Year FE	√	√	√	√
Sector FE	√	√	√	√
Province FE	√	√	√	√
Firm FE	×	×	√	√
Observations	103,650	101,417	93,391	91,475
R-squared	0.233	0.278	0.851	0.852

Note: Robust Standard errors in parentheses; *** 1%, ** 5%.

**Table 13:** Impact of the SO_2_ ETS on robot application.

Dep. Var.	FE		Heckman Two-Stage Model	
	(1) ln*RobotV*	(2) ln*RobotN*	(3) ln*RobotV*	(4) ln*RobotN*
*time × treat*	0.028 **	0.003 *	0.591 ***	0.076 ***
	(0.012)	(0.002)	(0.056)	(0.008)
*lambda*			4.008 ***	0.524 ***
			(0.377)	(0.055)
_cons	−0.130 **	−0.019 **	−19.879 ***	−0.049 ***
	(0.060)	(0.009)	(1.859)	(0.005)
Control variables	√	√	√	√
Year FE	√	√	√	√
Sector FE	√	√	√	√
Province FE	√	√	√	√
Firm FE	√	√	×	×
Observations	135,541	124,085	91,401	91,401
R-squared	0.014	0.396	0.019	0.012

Note: Robust Standard errors in parentheses; *** 1%, ** 5%, * 10%.

**Table 14:** Impact of robot application on total factor productivity of enterprises.

Dep. Var.	OLS				2SLS			
	(1) *Tfp_lp*		(2) *Tfp_op*		(3) *Tfp_lp*		(4) *Tfp_op*	
ln*RobotV*	0.036 ***		0.023 ***		4.556 **		8.159 **	
	(0.004)		(0.004)		(1.996)		(3.512)	
ln*RobotN*		0.199 ***	−0.039 ***	0.137 ***		28.815 **		51.607 **
		(0.020)	(0.002)	(0.020)		(11.852)		(20.773)
*Age*	−0.034 ***	−0.034 ***	−0.350 ***	−0.039 ***	0.087 ***	0.087 ***	0.115 **	0.114 **
	(0.003)	(0.003)	(0.017)	(0.002)	(0.029)	(0.026)	(0.052)	(0.046)
*Owner*	−0.324 ***	−0.324 ***	0.159 ***	−0.350 ***	−0.123	−0.286	−0.070	−0.361
	(0.019)	(0.019)	(0.005)	(0.017)	(0.149)	(0.246)	(0.264)	(0.439)
*Scale*	0.470 ***	0.470 ***	1.372 ***	0.160 ***	−0.022	0.019	−0.197 ***	−0.124 **
	(0.007)	(0.007)	(0.510)	(0.005)	(0.029)	(0.030)	(0.050)	(0.052)
*Kl*	1.948 ***	1.949 ***	0.000	1.372 ***	0.689 **	0.710 ***	0.800	0.837 **
	(0.734)	(0.734)	(0.001)	(0.510)	(0.294)	(0.255)	(0.528)	(0.387)
*Alr*	0.000	0.000	0.231 ***	0.000	0.000	0.000	0.000	0.000
	(0.001)	(0.001)	(0.041)	(0.001)	(0.000)	(0.000)	(0.000)	(0.000)
*Se_gdp*	0.335 ***	0.334 ***	−0.022 ***	0.231 ***	0.129	−0.136	−0.790	−1.264
	(0.043)	(0.043)	(0.002)	(0.041)	(0.515)	(0.578)	(0.904)	(1.011)
*Regulation*	−0.012 ***	−0.012 ***	0.023 ***	−0.022 ***	0.025 ***	0.024 ***	0.020	0.018
	(0.002)	(0.002)	(0.004)	(0.002)	(0.008)	(0.007)	(0.014)	(0.012)
Year FE	√	√	√	√	√	√	√	√
Sector FE	√	√	√	√	×	×	×	×
Province FE	√	√	√	√	×	×	×	×
Firm FE	×	×	×	×	√	√	√	√
Observations	129,900	129,900	129,900	129,900	117,977	117,977	117,977	117,977
R-squared	0.327	0.327	0.168	0.168	−14.595	−14.935	−45.769	−46.822

Note: Robust Standard errors in parentheses; *** 1%, ** 5%.

**Table 15:** Regional heterogeneity analysis.

Dep. Var.	(1) Resource-Based City	(2) Growing Resource-Based City	(3) Mature Resource-Based City	(4) Declining Resource-Based City	(5) Regenerative Resource-Based City
*time × treat*	−0.126 ***	0.469 *	−0.050	−0.390 ***	−0.122
	(0.014)	(0.253)	(0.067)	(0.150)	(0.125)
*Age*	0.028 ***	−0.026	0.027	0.021	0.005
	(0.003)	(0.052)	(0.017)	(0.031)	(0.022)
*Owner*	0.179 ***	0.482 *	0.148 *	0.174	−0.168
	(0.020)	(0.258)	(0.079)	(0.140)	(0.127)
*Scale*	−0.055 ***	−0.094 *	−0.050 ***	0.026	−0.056 **
	(0.003)	(0.053)	(0.016)	(0.035)	(0.026)
*Kl*	−0.385 ***	−0.080	−0.010	−9.713 ***	−0.013
	(0.080)	(0.220)	(0.008)	(3.576)	(0.017)
*Alr*	0.002 ***	0.043	0.287 ***	0.020	0.019
	(0.001)	(0.199)	(0.069)	(0.118)	(0.103)
*Se_gdp*	0.026	3.926 ***	−0.969 ***	1.042 *	0.602
	(0.059)	(1.308)	(0.304)	(0.631)	(1.211)
*Regulation*	−0.011 ***	−0.020	−0.052 ***	−0.028	0.022
	(0.003)	(0.034)	(0.009)	(0.017)	(0.029)
_cons	1.396 ***	−0.110	2.422 ***	0.994 **	1.431 *
	(0.037)	(0.758)	(0.189)	(0.414)	(0.774)
Year FE	√	√	√	√	√
Sector FE	√	√	√	√	√
Province FE	√	√	√	√	√
Observations	92,178	434	5624	1512	1954
R-squared	0.226	0.510	0.217	0.343	0.216

Note: Robust Standard errors in parentheses; *** 1%, ** 5%, * 10%.

## Data Availability

The data presented in this study are available on request from the authors.

## Appendix A

**Table A1:** Effect of emission trading policy on sulfur dioxide emissions.

Dep. var.	SO_2_	
	(1)	(2)
*time × treat*	−0.111 ***	−0.176 ***
	(0.008)	(0.007)
*Se_gdp*		2.273 ***
		(0.035)
*Invest*		0.000 ***
		(0.000)
*Em*		−0.000 ***
		(0.000)
*Pergdp*		−0.000 ***
		(0.000)
*Fdi*		0.000 ***
		(0.000)
_cons	11.145 ***	10.099 ***
	(0.003)	(0.032)
Control variables	×	√
Year FE	√	√
Province FE	√	√
Observations	103,650	101,417
R-squared	0.225	0.227

Note: Robust Standard errors in parentheses; *** 1%.

**Table A2:** Results of kernel matching balance test for propensity scores.

Variable	Unmatched Matched	Mean		% Bias	% Reduct Bias	*t*-Test	*p* > |*t*|
		Treated	Control				
*Age*	U	1.512	1.408	8.0	83.8	12.69	0.000
	M	1.512	1.494	1.3		1.85	0.065
*Owner*	U	0.060	0.053	3.1	89.2	4.90	0.000
	M	0.060	0.061	−0.3		−0.48	0.630
*Scale*	U	6.049	6.072	−2.0	65.3	−3.16	0.002
	M	6.049	6.041	0.7		1.04	0.300
*Kl*	U	0.010	0.012	−0.7	68.8	−0.99	0.320
	M	0.010	0.009	0.2		1.38	0.167
*Alr*	U	0.598	0.584	0.3	45.2	0.45	0.655
	M	0.598	0.590	0.2		0.22	0.829
*Se_gdp*	U	0.527	0.495	43.4	85.1	67.34	0.000
	M	0.527	0.532	−6.5		−10.88	0.000
*Regulation*	U	3.609	2.800	31.4	54.6	49.61	0.000
	M	3.609	3.241	14.3		20.51	0.000

**Table A3:** Results of radius matching balance test for propensity scores.

Variable	Unmatched Matched	Mean		% Bias	% Reduct Bias	*t*-Test	*p* > |*t*|
		Treated	Control				
*Age*	U	1.512	1.408	8.0	82.1	12.69	0.000
	M	1.512	1.494	1.4		2.04	0.041
*Owner*	U	0.060	0.053	3.1	90.7	4.90	0.000
	M	0.060	0.061	−0.3		−0.41	0.679
*Scale*	U	6.049	6.072	−2.0	67.0	−3.16	0.002
	M	6.049	6.041	0.7		0.99	0.323
*Kl*	U	0.010	0.012	−0.7	68.1	−0.99	0.320
	M	0.010	0.009	0.2		1.41	0.159
*Alr*	U	0.598	0.584	0.3	49.9	0.45	0.655
	M	0.598	0.591	0.2		0.20	0.845
*Se_gdp*	U	0.527	0.495	43.4	85.7	67.34	0.000
	M	0.527	0.532	−6.2		−10.46	0.000
*Regulation*	U	3.609	2.800	31.4	53.8	49.61	0.000
	M	3.609	3.237	14.5		20.88	0.000
